# An Ultrahigh-Sensitivity Graphene Resonant Gyroscope

**DOI:** 10.3390/nano11081890

**Published:** 2021-07-23

**Authors:** Yang Lu, Zhan-She Guo, Shang-Chun Fan

**Affiliations:** 1 School of Instrumentation Science and Opto-Electronics Engineering, Beihang University, Beijing 100191, China; Lu_yang@buaa.edu.cn; 2 Key Laboratory of Quantum Sensing Technology, Ministry of Industry and Information Technology, Beijing 100191, China

**Keywords:** ultrahigh sensitivity, graphene, resonance, gyroscope

## Abstract

In this study, a graphene beam was selected as a sensing element and used to form a graphene resonant gyroscope structure with direct frequency output and ultrahigh sensitivity. The structure of the graphene resonator gyroscope was simulated using the ANSYS finite element software, and the influence of the length, width, and thickness of the graphene resonant beam on the angular velocity sensitivity was studied. The simulation results show that the resonant frequency of the graphene resonant beam decreased with increasing the beam length and thickness, while the width had a negligible effect. The fundamental frequency of the designed graphene resonator gyroscope was more than 20 MHz, and the sensitivity of the angular velocity was able to reach 22,990 Hz/°/h. This work is of great significance for applications in environments that require high sensitivity to extremely weak angular velocity variation.

## 1. Introduction

Resonant gyroscopes with direct frequency output can directly convert a weak Coriolis angular velocity into a frequency-modulated wave by frequency modulation and demodulate a frequency-modulated wave to calculate the input angular velocity. Compared with the conventional amplitude modulation detection method, it has the characteristics of high precision, good stability, low power consumption, and small drift. It can also further improve the gyro test accuracy when new materials with excellent structural characteristics are selected. The birth of graphene materials makes it possible to develop a resonant gyroscope with a direct frequency output that is sensitive to ultraweak angular velocity variation.

**Figure 1:**
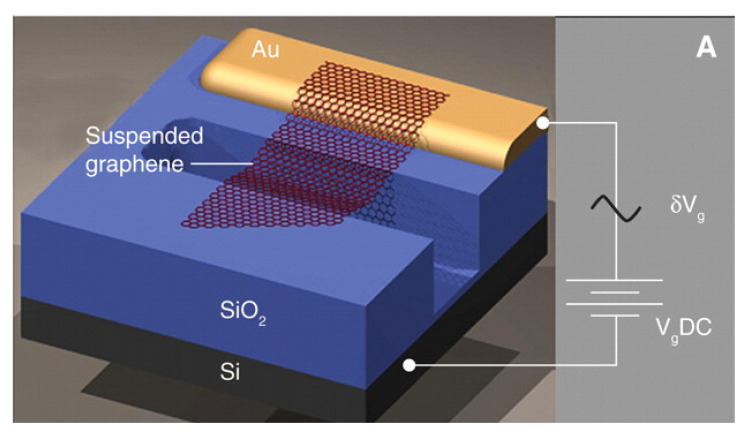
Prototype of a graphene resonator.

In 2004, the physicists Konstantin Novoselov and Andre Geim from the University of Manchester successfully peeled single-layer graphene from graphite for the first time [1]. Graphene has a Young’s modulus as high as 1 TPa and a tensile strength reaching 130 GPa—significantly higher than that of silicon (165 GPa and 5–9 GPa, respectively)—which is commonly used in microsystem sensors [2,3,4,5]. In 2007, Bunch et al. of Cornell University developed prototype nano-electromechanical resonators, using single- and multilayer graphene films, as shown in Figure 1 [6]. The monolayer or multilayer graphene films obtained by the mechanical peeling process were adsorbed onto the shallow grooves of the SiO_2_ insulating layer via intermolecular van der Waals forces to form double-clamped graphene beams. Gold electrodes set at both ends of the SiO_2_ layer allowed the resonator to vibrate within the MHz range upon electric excitation. The frequency of the single-layer graphene resonator was 70.5 MHz, and the quality factor (Q) was about 78. This experiment provided important support for the subsequent design and experiments using graphene resonators.

**Figure 2:**
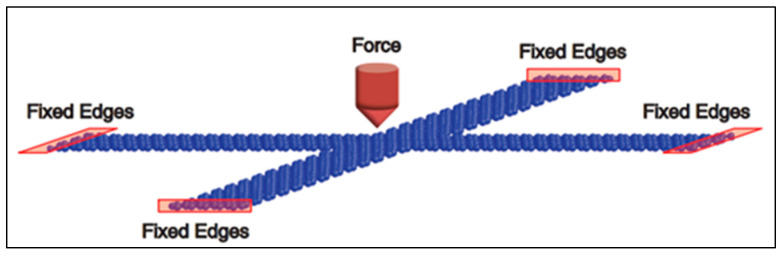
Accelerometer based on suspended cross-type graphene resonator.

**Figure 3:**
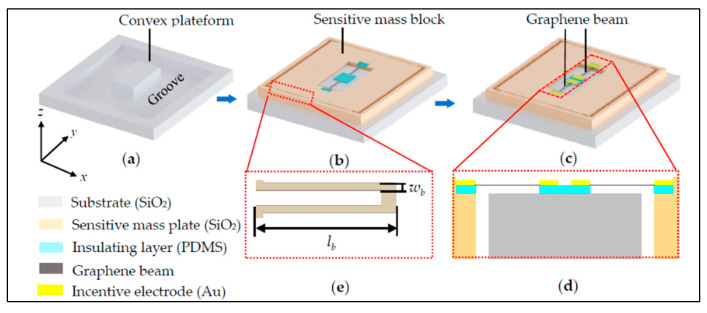
The model of a differential resonant graphene accelerometer: (**a**) the substrate of the accelerometer; (**b**) etching sensitive mass plate and preparing insulating layers and graphene beams; (**c**) fabricating the integrated accelerometer; (**d**) enlarged y–z cross-sectional view of the central area of accelerometer; (**e**) partial enlarged drawing of one side folded support beam in inset [12].

In 2009, Chen et al. of Columbia University developed a single-layer graphene resonator with an electrical readout. The research group experimentally tested the variation of the resonant frequency of the graphene resonator with the additional mass, external temperature, and voltage of the silicon electrodes [7]. In 2012, Kang et al. designed and studied an ultrahigh-sensitivity accelerometer based on graphene nanoribbons [8]. In 2013, Kwon et al. used molecular dynamics to study the relationship between the fundamental frequency of double-clamped graphene resonators and the additional mass [9]. In 2013, Natsuki et al. studied a graphene resonant nanomass sensor using continuous elastic theory in which a double-layer graphene diaphragm was used as the resonator [10]. In 2015, Kang et al. conducted a study on the dynamic characteristics of an accelerometer based on a suspended cross-type graphene resonator; its theoretical model is shown in Figure 2 [11]. In 2018, Shi Futao et al. of Beihang University designed a differential graphene resonance acceleration sensor, which is shown in Figure 3. In this paper, the Euler–Bernoulli beam model was used to analyze the double-clamped graphene beam of graphene resonant accelerometer. Through finite element simulation, the graphene resonant accelerometer with ultra-high sensitivity characteristics for detecting acceleration was obtained [12].

In summary, graphene resonators have been developed well in the fields of inertial navigation [8,11,12] and micro-mass detection [10,13]; however, research on graphene resonant gyroscopes for angular velocity measurement has not yet been reported.

On the basis of the principle of resonant gyroscopes for MEMS (Micro-Electro-Mechanical System)with direct frequency output and the model of graphene resonators, a graphene resonant gyroscope was designed. By simulating the influence of the length, width, and thickness of the graphene resonant beam on the resonant frequency of the gyroscope sensor, an optimized finite element simulation structure for the graphene resonant gyroscope was obtained.

## 2. The Operating Principle

### 2.1. Theoretical Basis of Resonant Gyroscope with Direct Frequency Output

The concept of resonance has been well applied in the drive and detection modes of resonant gyroscopes with direct output frequencies. Through detecting the change in the resonant frequency of the resonator, the input can be measured, which is an FM (frequency modulation) detection method. Unlike other MEMS sensors that detect weak capacitance analog signals, this FM detection method converts the weak Coriolis force into FM waves and then calculates the angular velocity load by demodulating the FM waves. Compared with the conventional amplitude modulation detection method, this detection method has high accuracy, a large dynamic range, low noise, and low power consumption. In 2002, the University of California, Berkeley, designed the first integrated micromechanical gyroscope with direct output frequency detection [14,15]; the research on resonant gyroscopes with direct output frequency has been continuously improved since then [16,17].

**Figure 4:**
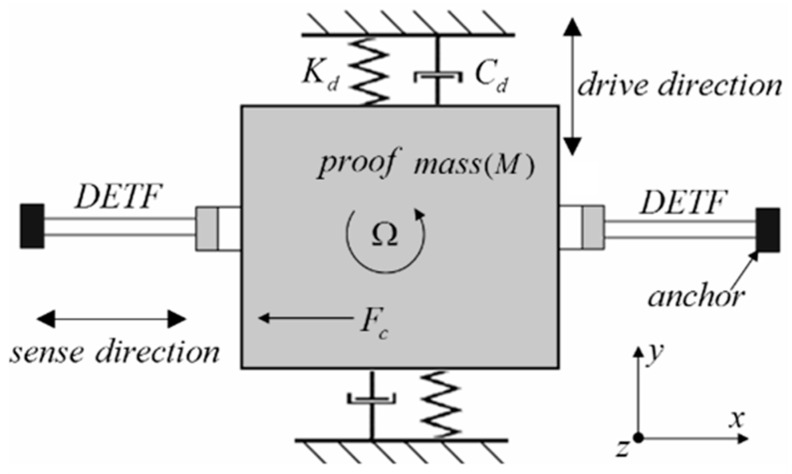
Schematic diagram of resonant gyroscope with direct frequency output.

As shown in Figure 4, the mass vibrates in the *y*-axis direction with its resonant frequency under the action of an external driving force, while the two axisymmetric resonators, DETF (double-end tine fork), vibrate in the reverse direction, with their resonant frequency under the action of the driving force. When the angular velocity along the *z*-axis is generated, according to the principle of the Coriolis effect, the Coriolis force is generated on the mass along the *x*-axis of the plane perpendicular to the *y*-axis of the relative motion direction and the *z*-axis of the angular velocity direction. The periodic pressure or tension generated by the driving mass acts on both ends of the DETF such that the resonant frequency of the DETF changes. The frequency variation of the DETF is proportional to the Coriolis force; thus, the loading angular velocity can be demodulated. Here, *y*_0_ is the maximum amplitude of the mass block, *y* is the displacement of the mass block in the *y*-axis direction, *w_d_* is the driving frequency of the mass block, *M* is the mass of the mass block, and *Ω* is the angular velocity that is set at a constant value. Combining the vector derivative with Newton’s second mechanical equation, the Coriolis force can be expressed as follows:

(1) $$F_{C}=2M\vec{\Omega }\times \vec{V_{B}}$$

*V_B_*, as the relative velocity of the mass block relative to the frame, can be expressed as follows:

(2) $$V_{B}=\dot{y}=\omega_{d}\cdot y_{0}\cdot cos\left(\omega_{d}t\right)$$

where

(3) $$y=y_{0}\cdot sin\left(\omega_{d}t\right)$$

Then, the Coriolis Force *F_c_* can be rewritten as the following:

(4) $$F_{c}=2M\Omega \omega_{d}y_{0}cos\left(\omega_{d}t\right)$$

### 2.2. Theoretical Analysis of Double-Clamped Graphene Resonant Beam

At present, there is no perfect theoretical model for the study of graphene resonance characteristics. The research methods described in the literature mainly include the traditional continuum model [18], the semi-continuum theory, and molecular dynamics simulations [10,19,20]. For single-layer or multilayer graphene, thin plates, shells, and other continuum or semi-continuum models are used to perform the modeling. Then, the bending, tension, vibration, and other mechanical behaviors and mechanical properties of graphene are analyzed, using the Euler beam and Timoshenko plate shell theories [21,22]. Numerical modeling can also be carried out using commercial finite element analysis software, such as COMSOL/ANSYS. In this study, the continuous medium model was used to analyze the double-clamped graphene resonant beam.

When analyzing the lateral bending free vibration of the beam, the following assumptions are first introduced:

(a) The central inertia axis of each section of the beam is in the same plane, and the beam moves laterally in this plane.
(b) The ratio of the cross-sectional area size of the beam to its length is relatively small, and the influence of shear deformation and the moment of inertia around the central axis of the section can be ignored.
(c) The transverse vibration of the beam conforms to the assumption of small deflection plane bending, i.e., the amplitude of the transverse vibration is very small and within the linear range.

**Figure 5:**
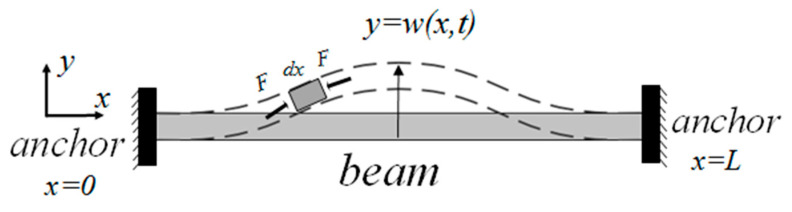
Bending model of graphene resonant beam subjected to force.

According to the above assumptions, the structural dimensions of the graphene resonance beam designed in this paper are shown in Table 1; the ratio of the cross-sectional area size of the beam to its length is relatively small ($0.11\times 10^{-9}$). This type of mechanical model, which only considers the lateral deformation caused by bending and does not consider the bending vibration of the beam due to shear deformation and the moment of inertia, becomes the Euler–Bernoulli beam. The forced vibration characteristics of the beams can be analyzed using the Euler–Bernoulli beam theory as shown in Figure 5 [23].

The length, width, and thickness of the graphene resonant beam are *L*, *b*, and *h*, respectively. The microelement d*x* was chosen as the object of study; y(x,t) is the deflection (lateral displacement) of the element at time *t*, and  F(x,t) is the external force exerted by the element d*x* at time *t*. The vibration equation of the graphene resonant beam can then be approximated as follows [24]:

(5) $$EI\frac{\partial^{4}y\left(x,t\right)}{\partial x^{4}}-T\frac{\partial^{2}y\left(x,t\right)}{\partial x^{2}}+\rho A\frac{\partial^{2}y\left(x,t\right)}{\partial t^{2}}=F\left(x,t\right),$$

where *ρ* is the density of graphene, *A* is the cross-section area of the beam (i.e., *A* = *bh*), *E* is the Young’s modulus of the beam, *I* is the moment of inertia of the cross-section of the beam to the neutral axis (i.e., *I* = *bh*^3^/12), and *T* is the axial force acting along the *x*-direction of the resonant beam in the units N. The solution of the equation is given as follows:

(6) y(x,t)=y(x)cos(ωt),

where *ω* is the natural frequency of the resonant beam in rad/s. Combining Equation (6) with Equation (5), the mode function becomes the following:

(7) $$y\left(x\right)=A\;sin\left(\lambda_{1}x\right)+B\;cos\left(\lambda_{1}x\right)+C\;sinh\left(\lambda_{2}x\right)+D\;cosh\left(\lambda_{2}x\right).$$

According to the boundary condition of the double-clamped beam, the natural frequencies of double-clamped beams under axial force *T* can be derived. The first-order bending vibration frequency is given by the following:

(8) $$f\left(T\right)=f_{0}\sqrt{1+0.2949\frac{TL^{2}}{Ebh^{3}}}=f_{0}\sqrt{1+0.2949\varepsilon_{x}{\left(\frac{L}{h}\right)}^{2}},$$

where *f*_0_ represents the first natural frequency when the axial force *T* = 0, and *ε_x_* represents the axial strain of the beam as follows:

(9) $$\;f_{0}=\frac{4.730^{2}h}{2\pi L^{2}}\sqrt{\frac{E}{12\rho }},\;\varepsilon_{x}=\frac{T}{Ebh}.$$

## 3. Design and Simulation of Graphene Resonator Gyroscope

**Figure 6:**
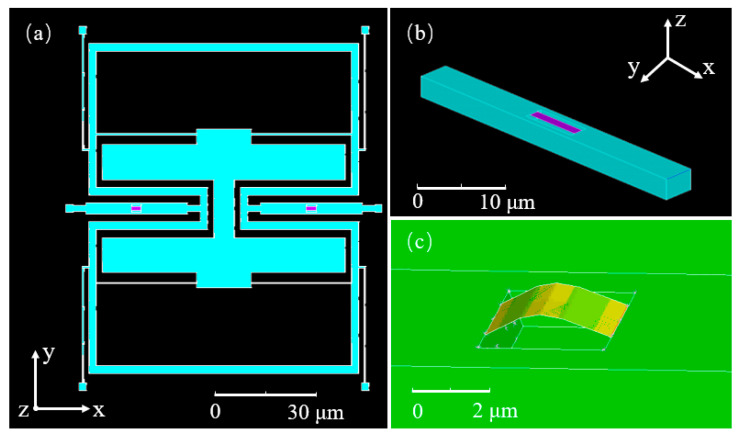
Finite element simulation model of graphene resonant gyroscope and graphene resonant beam: (**a**) the driving mode of the graphene resonant gyroscope; (**b**) double-clamped graphene beam supported on a Si substrate; (**c**) the detecting mode of graphene resonator gyroscope.

On the basis of the principle of resonant gyroscopes with direct frequency output described in Figure 4 of Section 2.1, an ANSYS simulation model of a graphene resonant gyroscope was established as shown in Figure 6a. The working principle of the graphene resonator gyroscope is as follows:

(a) On the basis of silicon-based materials, single graphene beams are supported by intermolecular van der Waals forces in the etched grooves of the Si transfer beams, as shown in Figure 6b.
(b) The mass block is driven to move in a simple and harmonic way in the *y*-axis direction. When the angular velocity in the *z*-axis direction is generated, as stated in Equation (1), the Coriolis force is generated in the *x*-axis direction.
(c) The Coriolis force passes through the *x*-axis direction of the symmetrical Si transfer beam, which causes the Si transfer beam to generate axial strain, which in turn causes an axial stress change in the double-clamped graphene beam on the Si transmission beam, effectively changing the resonant frequency state of the graphene.
(d) As stated in Equation (4), the magnitude of the angular velocity is demodulated by tuning the direct output frequency.

The axially symmetrical graphene beams are stretched and compressed in the working state, and the reciprocating motion realizes differential detection, which reduces the common mode interference. The resonant frequency of graphene is very high, which can respond to changes in the small Coriolis force, thus realizing ultrahigh sensitivity. Figure 6 shows the ANSYS simulation mode detection of the graphene resonator gyroscope. The first mode of the graphene resonator gyroscope is the driving mode shown in Figure 6a, while the second mode of the graphene resonator gyroscope shown in Figure 6c represents the dual graphene resonator beam entry frequency detection.

A graphene resonant beam is the core sensitive element of a resonant sensor, so its dynamic characteristics are very important. Since the thickness of a single graphene layer is only 0.335 nm—much lower than the minimum meshing size in the ANSYS finite element software—it is not possible to directly use the “Solid” module in the software for modeling. In this study, a rectangular graphene resonant beam structure was constructed using the “beam” or “shell” provided in the software. Its thickness value was directly set in the setup window, which simplified the geometric modeling process and meshing, in addition to improving the efficiency of solving. The thickness of all silicon structures is 3um. The geometric parameters and material properties of the silicon base and graphene resonator beams are shown in Table 1. Furthermore, the MPC184 model was chosen, and a multipoint constraint connection was used, as shown in Figure 6b.

Residual stress is generated during the preparation of graphene materials and the transfer of graphene materials to the Si substrate. Such residual stress has a great influence on the detection of the resonant frequency of the graphene material; hence, it is necessary to optimize the process during the preparation and transfer process to reduce the influence of the residual stress. At the same time, due to the influence of the processing technology, the residual stress of the graphene materials produced by each batch of processing is not completely identical, and the influence of the residual stress can be reduced but cannot be completely eliminated. In this study, we minimized the effect of residual stress on the resonant frequency of graphene, and we simulated and analyzed the situation where the most ideal stress was 0. To verify the validity of this method, experimental data from published papers were compared, as shown in Table 2. The error was within the allowable controllable range, thus proving the feasibility of this method.

**Figure 7:**
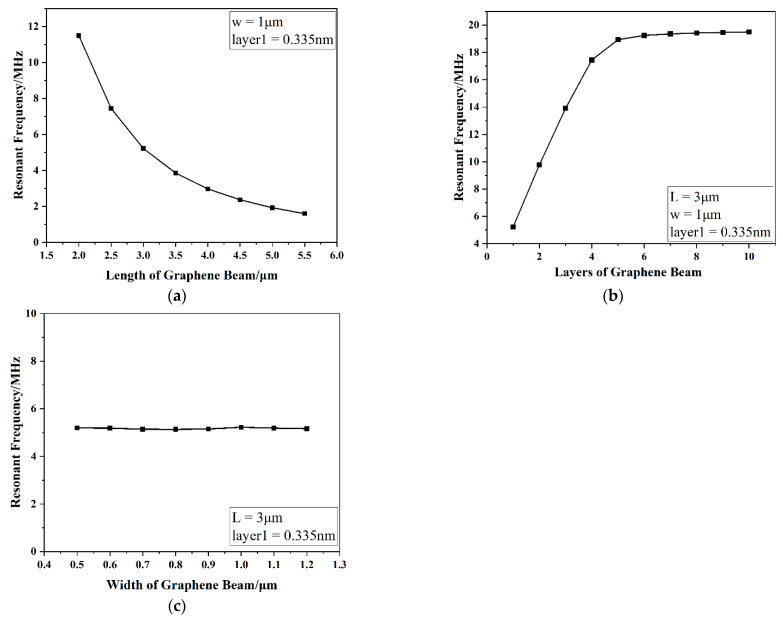
Influence of the graphene resonant beam structure size on the frequency output: (**a**) the relationship between the length of the graphene beam and resonant frequency; (**b**) the relationship between the thickness of the graphene beam and resonant frequency; (**c**) the relationship between the width of the graphene beam and resonant frequency.

Using the above model, the influence of the structure parameters of graphene resonator beams on the angular velocity sensitivity was studied. In Figure 7a, the resonant frequency of a 2–5.5 μm-length graphene beam was simulated with a fixed width of 1 μm and thickness of 0.335 nm. With increasing length, the resonant frequency decreased, the sensitivity of the resonator decreased, and the degree of nonlinearity decreased. The resonant frequency of a graphene beam with a fixed length of 3 μm and width of 1 μm was simulated, and the results are shown in Figure 7b. Here, the thickness increased from one layer (0.335 nm) to 10 layers, and the resonant frequency of the beam increased gradually under the same load, while the sensitivity gradually decreased. The linearity of layers 1–5 was good, and the change in the resonant frequency of layers 6 and above was relatively gentle, in contrast to the Euler–Bernoulli beam theory. This trend may be caused by a variety of reasons, and it will be further explored. As shown in Figure 7c, the width of the graphene beam was varied with a fixed length of 3 μm and thickness of 0.335 nm. At different widths, the curves of the uniform load and the resonant frequency of the beam almost coincided, indicating that the width of the graphene beam had little influence on the sensitivity of the resonator.

**Figure 8:**
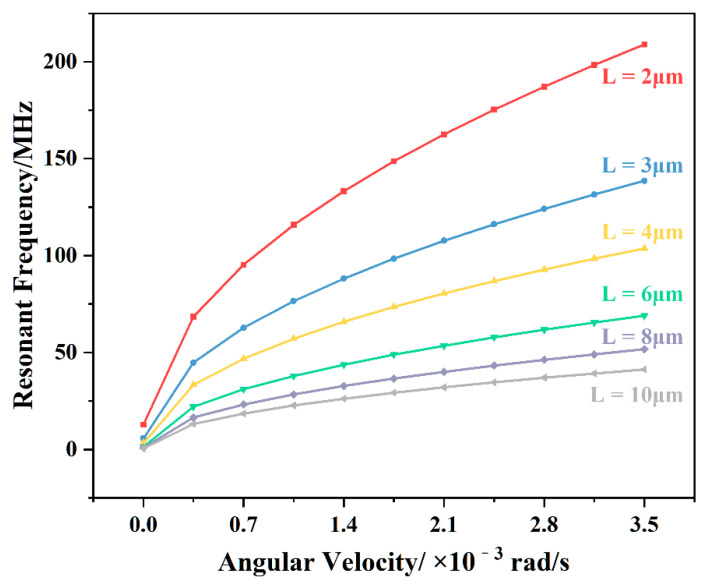
Curves of the resonant frequencies of graphene resonance beams of different lengths against the Coriolis force.

**Figure 9:**
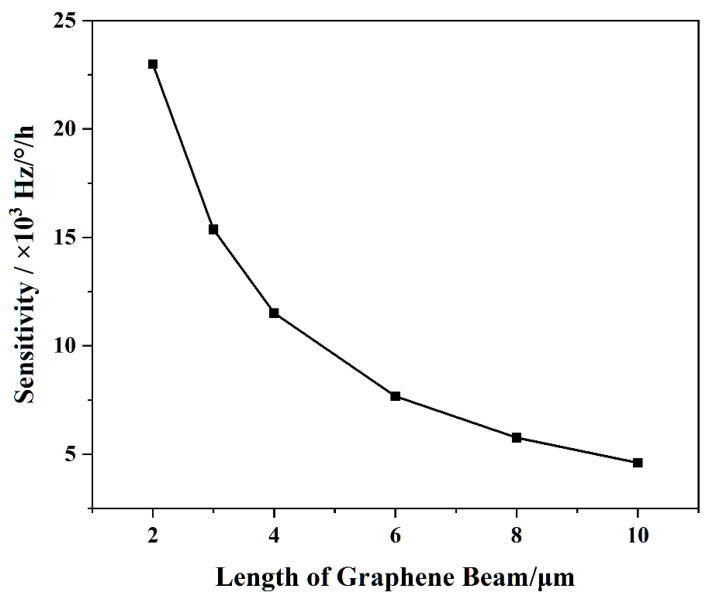
Sensitivity versus length of the graphene resonant beam.

Through the simulation analysis of the angular velocity sensitivity of the geometric size of the graphene resonator beam, it was found that the length change in the graphene resonator beam had a greater influence on the angular velocity sensitivity. When the angular velocity was converted to the Coriolis force acting on the graphene resonator beam, with $M=1.058\times 10^{-11\;}\mathrm{kg}$, $\omega_{d}=$ 22431 Hz, $\;y_{0}=3.15\;\mu m$, the results of the simulation and using Equation (4), the curve of the resonant frequency of the graphene resonator beam with different lengths under angular velocity was obtained as shown in Figure 8. The resonant frequency of the graphene resonator beam with different lengths showed the same trend with an increase in angular velocity, that is, the slope decreased, and the sensitivity decreased. As shown in Figure 9, the angular velocity sensitivity significantly decreased with the increasing length of the graphene resonator. Under the structural conditions of length = 2 μm, width = 1 μm, and thickness = 0.335 nm, the graphene resonant gyroscope had a sensitivity of up to 22,990 Hz/°/h.

## 4. Conclusions

In this study, a graphene resonant gyroscope structure with a graphene beam resonator was designed. Theoretical analyses and finite element simulations of the graphene resonant beam structure and the graphene resonant gyroscope were carried out. The fundamental frequency of the graphene resonant gyroscope was determined to be more than 20 MHz, and the sensitivity of the angular velocity was able to reach 22,990 Hz/°/h. It was found that the length and thickness of the graphene resonant beam both affected the sensitivity of the sensor. The sensitivity decreased upon increasing both the length and the thickness of the graphene resonant beam. This work provides a theoretical basis for the design of an ultrahigh-sensitivity graphene resonator gyroscope that is sensitive to very weak angular velocity variation.

## Figures and Tables

**Table 1:** Initial parameters and material properties of silicon and graphene resonant beams.

**Si Geometric Parameters**			**Si Material Properties**		
Length (μm)	Width (μm)	Thickness (μm)	Young’s modulus (GPa)	Poisson ratio	Density (kg/m^3^)
30	3	3	130	0.28	2330
**Graphene Geometric Parameters**			**Graphene Material Properties**		
Length (μm)	Width (μm)	Thickness (nm)	Young’s modulus (GPa)	Poisson ratio	Density (kg/m^3^)
3	1	0.335	1000	0.16	2200

**Table 2:** Comparison of experimental data and finite element simulation.

Size (Length, Width, and Thickness)	Resonant Frequency of Experimental Data (MHz)	Resonant Frequency of Finite Element Simulation (MHz)	Error
1.1 μm× 1.93 μm × 0.3 nm	5.4 [6]	5.4983	1.82%
2.8 μm × 0.5 μm × 6 nm	17 [25] (The first frequency)	16.933	0.39%
	46 [25] (The second frequency)	46.992	2.1%
2.8 μm × 0.3 μm × 11 nm	31 [25]	30.986	0.04%

## Data Availability

The data are included in the main text.
